# Parsing Through the Data on Achilles Tendon Rupture Management, Rehab and Sports Return Criteria: a Current Literature Review

**DOI:** 10.1007/s12178-026-10043-w

**Published:** 2026-07-01

**Authors:** Lambert T. Li, Ansh Shah, Greta Wilker, Morgan W. Rice, Shana Miskovsky

**Affiliations:** UH of Cleveland Department of Orthopaedic Surgery, University Hospitals Drusinsky Sports Medicine Institute, Case Western Reserve University School of Medicine, Cleveland, OH USA

**Keywords:** Achilles tendon rupture, Achilles tendon reconstruction, Functional Achilles rehabilitation, Minimally invasive Achilles repair, Surgical repair, FHL transfer

## Abstract

**Purpose:**

To evaluate and discuss controversies that have arisen as management of acute and chronic Achilles tendon ruptures continues to evolve. Areas of debate include operative versus nonoperative treatment, best repair or reconstruction techniques, timing of post-op weightbearing protocols, and newer functional rehabilitation interventions. With the incidence of Achilles tendon ruptures increasing, the goal of this review is to provide a resource in determining best treatment options.

**Recent findings:**

Evidence demonstrates that operative and nonoperative management yield comparable long-term functional outcomes. However, surgical repairs offer lower rerupture rates and earlier return to activity. There is a trend towards minimally invasive techniques for primary repairs, favored due to reduced wound complications and faster recovery. Earlier surgical intervention after injury shows improved functional outcomes. For chronic ruptures, flexor hallucis longus tendon transfer is the workhorse of secondary reconstruction. Early functional rehabilitation has been shown to be safe and effective across treatment strategies.

**Summary:**

The management of Achilles tendon injuries remains a complex clinical challenge that demands a comprehensive understanding of the tendon biomechanics, and distinct pathophysiological differences between acute and chronic presentations. Selection between operative and non-operative management requires individualized consideration, while adhering to evidence-based rehabilitation protocols is paramount to successful functional outcomes. Navigating these patient specific treatment options for various patient populations promotes optimal recovery in the short and long term.

**Supplementary Information:**

The online version contains supplementary material available at 10.1007/s12178-026-10043-w.

## Introduction

The Achilles tendon transmits powerful forces generated by the gastrocnemius and soleus, and can withstand loads up to 8–11 times body weight during running [1]. Despite its strength, Achilles tendon rupture (ATR) has been increasing over recent years [2]. Sports participation is the most common mechanism of injury regardless of gender, with basketball possessing the highest for men and tennis for women [1, 3]. While athletic male populations aged 30–49 continue to have the highest rates of rupture, recent epidemiologic trends demonstrate important demographic shifts among women and the elderly. Namely, over the last decade, a Swedish study noted a 22% rise in incidence of ATR in women as compared to 17% for men [3]. At the same time, ATR is also increasing in frequency among the elderly. From 2015 to 2019, the incidence proportion increased in age groups 65–69, 70–74 and 75–79 [1]. This rise is likely linked to the growing popularity of recreational sports accessible to this population, such as Pickleball where the incidence of foot and ankle injuries has increased 6.5-fold from 2019 to 2023, with 39.4% of those injuries involving ATR with mean age at injury of 58.3 years [4].

The unique biomechanical demands of the Achilles tendon contribute to its susceptibility to rupture. The majority of the tendon is composed of type I collagen fibers which impart high tensile strength [5], while type 3 collagen provides initial structural support during injury healing but is less resistant to tensile forces. The extracellular matrix of the tendon is made up of elastin, fibronectin and proteoglycans that help maintain architecture under mechanical load and modulate injury response^5^. The middle region of the tendon (2–6 cm proximal to the calcaneal insertion) is the location of 80–88% of ruptures [5]. Due to hypovascularity, this region is more prone to hypoxia and higher temperatures which creates an environment of increased elasticity and reduced fiber strength [5].

Achilles ruptures typically occur due to forceful, eccentric muscular contraction during ankle plantarflexion from pushing off or from sudden ankle dorsiflexion from landing. There are multiple extrinsic and intrinsic factors that can predispose to rupture [5]. Exercise program errors that result in an abrupt increase in mechanical loading of the tendon related to running mileage, training surface change (uneven or incline in particular), or high intensity interval training, are shown to lead to tissue structural damage [6]. Meanwhile, lack of gastrocsoleus strength and flexibility represent modifiable risk factors. Intrinsic factors related to patient anatomy and altered biomechanics include pes cavus, hyperpronation, leg length discrepancy, and neurogenic foot deformities. Importantly, systemic conditions (diabetes, end stage kidney disease, hyperparathyroidism, inflammatory arthropathies, gout) and adverse drug effects (corticosteroids, fluoroquinolones, bisphosphonates) raise susceptibility to ATR [7]. Finally, genetic factors such as different gene polymorphisms have been linked to rupture risk [5]. G1023T on chromosome 17 and MMP3 on chromosome 11 have been linked.

The management of acute and chronic achilles tendon ruptures is continually evolving. With progress, debate in the literature has arisen regarding indications for operative versus conservative treatment, optimal repair or reconstruction techniques (acute versus chronic ruptures), timing of post-op weightbearing protocols, and newer functional rehabilitation interventions. Given the increasing incidence of ATR, we aim to provide an overview of the clinical management and rehabilitation in determining the optimal treatment option for each patient.

## Acute Versus Chronic Ruptures

Achilles tendon ruptures are generally classified as either acute or chronic. Acute ruptures are typically diagnosed within 6 weeks of injury. Patients typically present after an acute injury describing a “pop” followed by pain and difficulty with plantarflexion. Physical exams often demonstrate a positive calf squeeze test (Thompson test-absence of passive plantarflexion with squeezing of the gastrocnemius) and loss of resting tension of the tendon (increased resting ankle dorsiflexion with patient prone and ipsilateral knee bent 90 degrees).

Chronic Achilles tendon ruptures (CATR) are defined as injuries in which the symptoms persist beyond 6 weeks from the initial injury. Often these cases result from missed diagnosis or delayed presentation and comprise of approximately 25% of ATRs [8]. Patients with chronic ATRs often present with chronic discomfort, leg fatigue, tendon deformity, calf atrophy, impaired push off strength, difficulty with single leg heel raise, and gait abnormalities (calcaneal gait, toe out gait). These findings may be more subtle than in the acute setting due to scar tissue formation. MRI imaging may serve as a diagnostic adjunct for assessing tendon integrity and assist with surgical planning by determining the extent of gap formation and tendinopathy.

The timing of surgical intervention on patient outcomes is critical. Hewitt et al. evaluated outcomes based on time from rupture to surgery: acute (0–6 days), subacute (7–13 days), delayed (14–41 days) and chronic (42 + days).^9^ Patients either had percutaneous repairs or open repairs. Complications were generally similar across groups and between surgical procedures with respect to deep vein thrombosis, nerve injury, wounds, and neuropathic pain. The overall rerupture rate was 3.7% with most occurring within 80 days after repair, with the subacute group possessing no reruptures. Patient reported outcomes were collected using the Patient-Reported Outcomes Measurement Information Systems Physical Function (PROMIS PF), PROMIS Pain interference (PI), as well as Foot and Ankle Single Assessment Numeric Evaluation (FA SANE). PROMIS PF, which assesses a person’s ability to do activities of daily living, was the only outcome that showed statistical significance between time groups, with the highest scores in those who underwent surgery within a week. Another study with 10-year follow-up after plantaris-augmented open repairs for acute and chronic ruptures concluded that there were no significant differences in outcome scores (American Orthopaedic Foot and Ankle Society Score, Victorian Institute of Sport Assessment- Achilles Scale, patient satisfaction) [10]. However, patients with chronic ruptures exhibited statistically significant gait abnormalities including reduced peak plantar pressure (*p* = 0.044), impaired balance (*p* = 0.003), and prolonged heel strike (*p* = 0.021).

While delayed repairs or chronic rupture can achieve comparable patient reported outcomes, there remains a difference in biomechanical and rerupture outcomes [8, 9]. Therefore, early diagnosis and timely management should remain a high priority for clinicians when assessing for ATR.

## Operative Versus Nonoperative Management Considerations

The optimal treatment of ATR continues to be an area of controversy and debate. Recommendation to pursue surgical repair (open or minimally invasive) or nonsurgical management is based on a shared decision-making process with the patient including a detailed discussion regarding their activity expectations, level of function required, health status, and rupture characteristics. Operative treatment is generally favored in young, active patients, with literature reporting a statistically significant lower rerupture rate, earlier return to work (average, 19 days earlier), higher rate of return to sport, less calf atrophy, and 18% greater gastrocnemius strength return at 18 months [11, 12]. Surgical intervention is indicated if the rupture is chronic and symptomatic, tendon is ruptured off of the attachment on the calcaneus, or in proximal ruptures with >1 cm of proximal retraction [11]. The increased rate of complication related to operative intervention is driven by wound related issues and sural nerve injury, which are decreased using minimally invasive techniques [12]. In contrast, nonoperative management is appropriate and considered in the following situations: older or more sedentary individuals with lower functional demands, patients with medical comorbidities that are detrimental to wound healing and raise risk for poor outcomes (e.g. uncontrolled diabetes, peripheral vascular disease, smoking, morbid obesity), patients with partial tears, patients who are still able to actively plantarflex their ankle, and patients with tendon gap less than 1 cm [9]. In patients who undergo conservative management, the tendon gap fills in with less robust tissue which contributes to a higher rerupture rate, more significant tendon elongation resulting in loss of push off power, and a chronic “sunken in” deformity of the Achilles.

In 2022 Myhrvold et al. published a multicenter randomized controlled trial comparing nonoperative treatment, open repair, and minimally invasive surgery in adults with Achilles tendon rupture [13]. When comparing rerupture rates, open repair had a rerupture rate of 0.6% compared to 6.2% for nonoperative management. For nerve injury, nonoperative patients had a 0.6% rate compared to 2.8% for open repair and 5.2% for minimally invasive repair. Deep infection rates were similar in surgical groups (below 2%), but superficial infection rate was 1.7% for open repair and 0.6% for minimally invasive repair. A key finding in this paper was that there was no significant difference in functional outcomes between management groups (*p* = 0.57). Achilles Tendon Total Rupture Score is based on 10 questions answered by patients on scale from 0 (major limitations) to 10 (no limitations) that was used to assess symptoms (pain, strength, stiffness, or fatigue), and physical function (limitations with activities of daily living, walking on uneven surfaces, walking quickly up the stairs or hill, running or jumping, or manual labor). When comparing management options, operative treatment provides the best protection against rerupture, although non-operative is favorable in avoiding nerve injury. Both surgical and non-surgical management have similar functional recovery at 1 year.

Overall, both management options yield comparable functional outcomes and can be viable options when selected appropriately for each individual patient. Operative treatment may reduce rerupture risk and facilitate earlier activity in young individuals, while nonoperative management remains reasonable for older or lower demand individuals.

## Nonoperative Management of Achilles Tendon Ruptures

Amongst nonoperative protocols, the mainstay is early functional rehabilitation. Regardless of the specific protocol used, the goal of nonoperative treatment is to encourage early tendon gliding and prevent formation of deep adhesions. Protocols generally begin with passive range of motion and advance to active motion and tensile loading of the tendon. The Glazebrook protocol is commonly used [14]. Criteria for this protocol include diagnosis within 2 days of injury and initiation of treatment within 7 days, midsubstance Achilles rupture confirmed via MRI, and patient’s ability to comply with the protocol. For the first 2 weeks, patients are immobilized in a plaster cast in maximum plantarflexion with no weight bearing. Partial weight bearing begins at 2 weeks and progresses until full weight bearing is achieved at 6 weeks. Active motion is started at 2 weeks, and patients are transitioned from a plaster cast to a walking boot with heel lifts. Heel lifts are gradually decreased in size starting at 6 weeks and closed and open chain kinetic exercises are started. At 8 weeks, the patient is weaned from the boot. Return to non-cutting or jumping exercise is allowed at 6 months once a patient has regained 80% strength and return to full sport is allowed at 12 months if the patient has regained 100% strength.

A 2019 meta-analysis identified 174 studies of nonoperative Achilles rupture protocols from 1979 to 2018 and evaluated the interventions used and outcomes measured [15]. It was found that the median time to weightbearing and exercise onset across these studies was 2 weeks. Newer studies tended to recommend a delay in weight bearing after injury, whereas older studies recommended initiation of immediate weight bearing.

## Operative Management of Achilles Tendon Ruptures

### Primary Repair

**Fig. 1:**
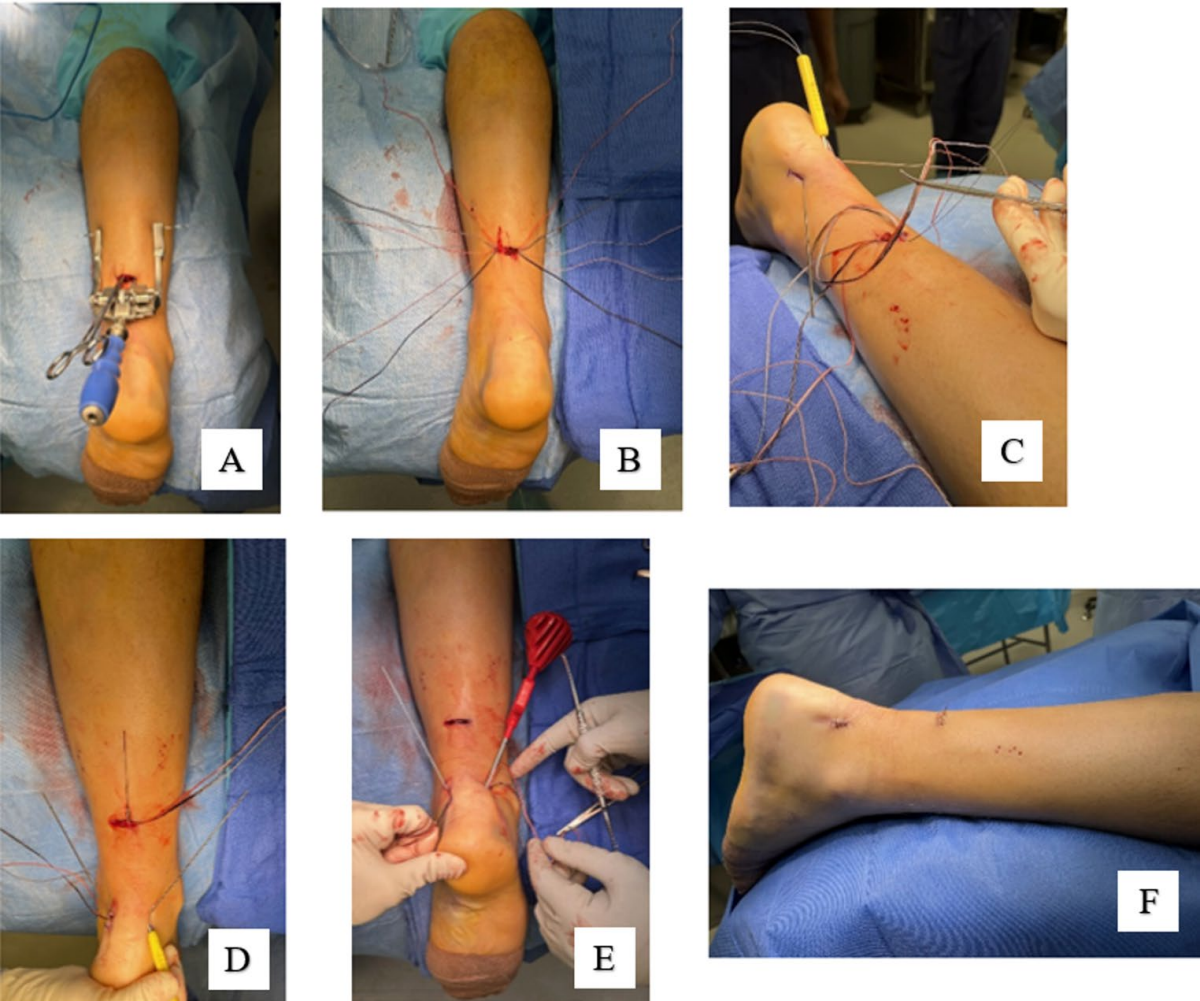
Example of hybrid percutaneous technique for primary achilles tendon repair. A small transverse incision is placed at or just above the level of the proximal Achilles stump. The paratenon is identified, released, and tagged for later repair. The sural neurovascular bundle is seen just lateral to edge of paratenon and gently protected with Crile retractor. An Allis is used to grasp the proximal stump and adhesions are released. (**A**) The arms of a specialized jig are placed within the paratenon tunnel and advanced around the tendon substance. Keith needles are placed through the jig to pass nonabsorbable sutures and two passing sutures through the proximal tendon stump. (**B**) Medial and lateral sutures are organized and laid out, and passing sutures are used to create medial and lateral krackow stitches. (**C**) Small posterolateral and posteromedial heel incisions are made, hugging the border of the Achilles laterally and medially. Blunt dissection down to the superior calcaneal tuberosity area is performed. A small calcaneal tunnel is drilled and tapped in preparation for anchor placement through each heel incision. A curved needle is placed through each heel incision, passed through the distal stump, and delivered out the transverse wound. (**C **and **D**). A nitinol loop is passed through the needle and the repair sutures are shuttled out the corresponding heel incision on each side. (**E**). The ankle is placed in 20 degrees of plantarflexion and the medial sutures are tensioned. The lateral sutures are passed through a suture anchor, tensioned, and seated into the pre-made tunnel and interference screw is advanced into calcaneus to fixate. Steps are repeated for the medial sutures. (**E**). Final construct shown after paratenon repair and wound closure

Primary, end-to end repair, can be accomplished through conventional open, minimally invasive surgery (MIS), and hybrid techniques. MIS techniques include both suture bridge constructs such as the Percutaneous Achilles Repair System (PARS; Arthrex, Inc., Naples, FL) or Achillion (Integra, Plainsboro, NJ, US) and artificial ligament augmentation constructs such as the Ligament augment reconstruction system (LARS). The authors’ preferred minimally invasive hybrid surgical technique is shown in Fig. 1 using a PARS jig proximally and suture anchors distally. Numerous recent studies have compared outcomes with conventional open techniques to minimally invasive techniques. Attia el al performed a meta-analysis of randomized controlled trials (RCTs), pooling results from 10 RCTs including a total of 522 patients [16]. The authors found no difference in respect to total complication rate and re-rupture rate. However, the open group had a superficial infection rate of 6.0% compared to 0.4% for MIS (*p* < 0.001). MIS repair was associated with shorter surgical time (29.7 versus 51.0 min, *p* < 0.001). Notably, there was a higher risk of temporary sural nerve palsy in the MIS group (3.4% versus 0%, *p* = 0.02).

Surgical technique selection, specifically primary repair versus FHL transfer, is another controversy. Chronicity of injury, tendon quality and size of the tear are all considerations. Primary end-to-end repair is typically indicated for acute ruptures in highly active individuals when tension free re-approximation of tendon stumps can be achieved. In a small number of chronic cases, primary repair may still be feasible if the tendon gap is less than 2 cm and soft tissue adhesions can be successfully released at surgery. The success of primary repair depends on adequate tendon mobilization and preservation of tissue integrity.

The FHL transfer is preferred in acute cases with larger soft tissue defects between 3 and 6 cm and poor remaining tissue quality [17]. Karaismailoglu et al. compared outcomes between primary repair and FHL transfer in acute ruptures and found no significant difference in ATRS, AOFAS scores, ankle plantarflexion strength or Tegner activity scores [18]. However, FHL transfer did demonstrate a greater percentage of patients that had returned to normal activities at the 9-month mark (91% vs. 73%, *p* < 0.01). This occurred with similar rates of overall complications at 10.3% in FHL vs. 13.6% in primary repair. Overall, both FHL tendon transfer and primary repair demonstrate favorable outcomes when appropriately selected.

**Fig. 2:**
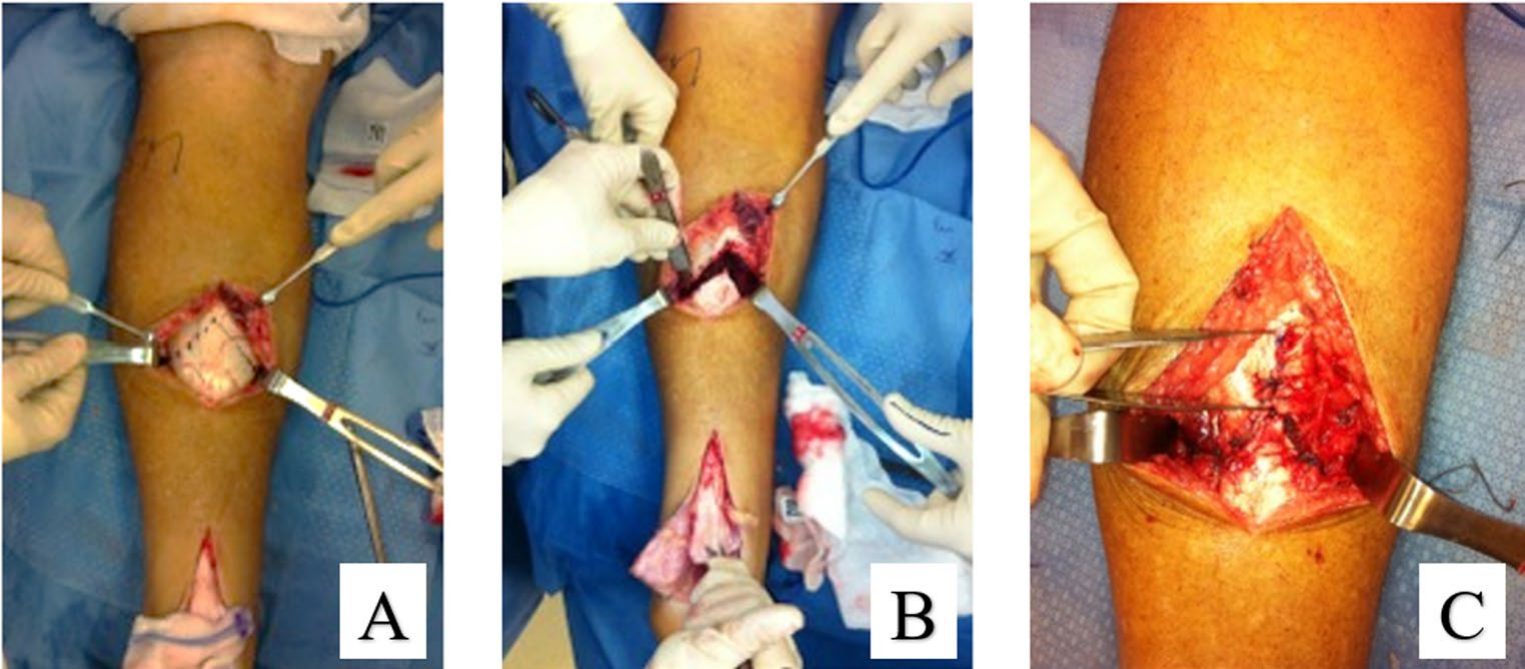
Proximal V-Y lengthening technique. A posterior longitudinal incision over the musculotendinous junction is made, sural nerve gently retracted, and superficial fascia released. (**A**) Each arm of the “V” created in the gastrocnemius aponeurosis is 1.5 times the length of the gap (in cm), with the apex of the “V” directed toward the patient’s head. (**B**) 11-blade is used to cut aponeurosis only, with the deeper white fascial bands seen in the cuts released carefully with tenotomy scissors. The V-shaped flap is pulled distally, creating a Y-shape. (**C**) The proximal vertical portion of the “Y” represents the amount of lengthening, set with side-to-side repair using nonabsorbable sutures. The arms of aponeurosis are repaired with Krackow stitches (proximal and distal on medial and lateral sides) and suture pairs are tied down with ankle in slight plantarflexion. Direct coaptation of those aponeurosis edges should not occur, otherwise lengthening is negated

Several useful adjuncts exist to aid in primary repair if there is a soft tissue gap or concern for increased tension on the primary repair. These include proximal V-Y lengthening [18] of the Achilles tendon **(**Fig. 2**)** and aponeurosis turndown flap [19]. These are generally utilized in the setting of chronic ruptures, when over 2 cm of tendon retraction is present.

### Secondary Reconstruction With Tendon Transfer

**Fig. 3:**
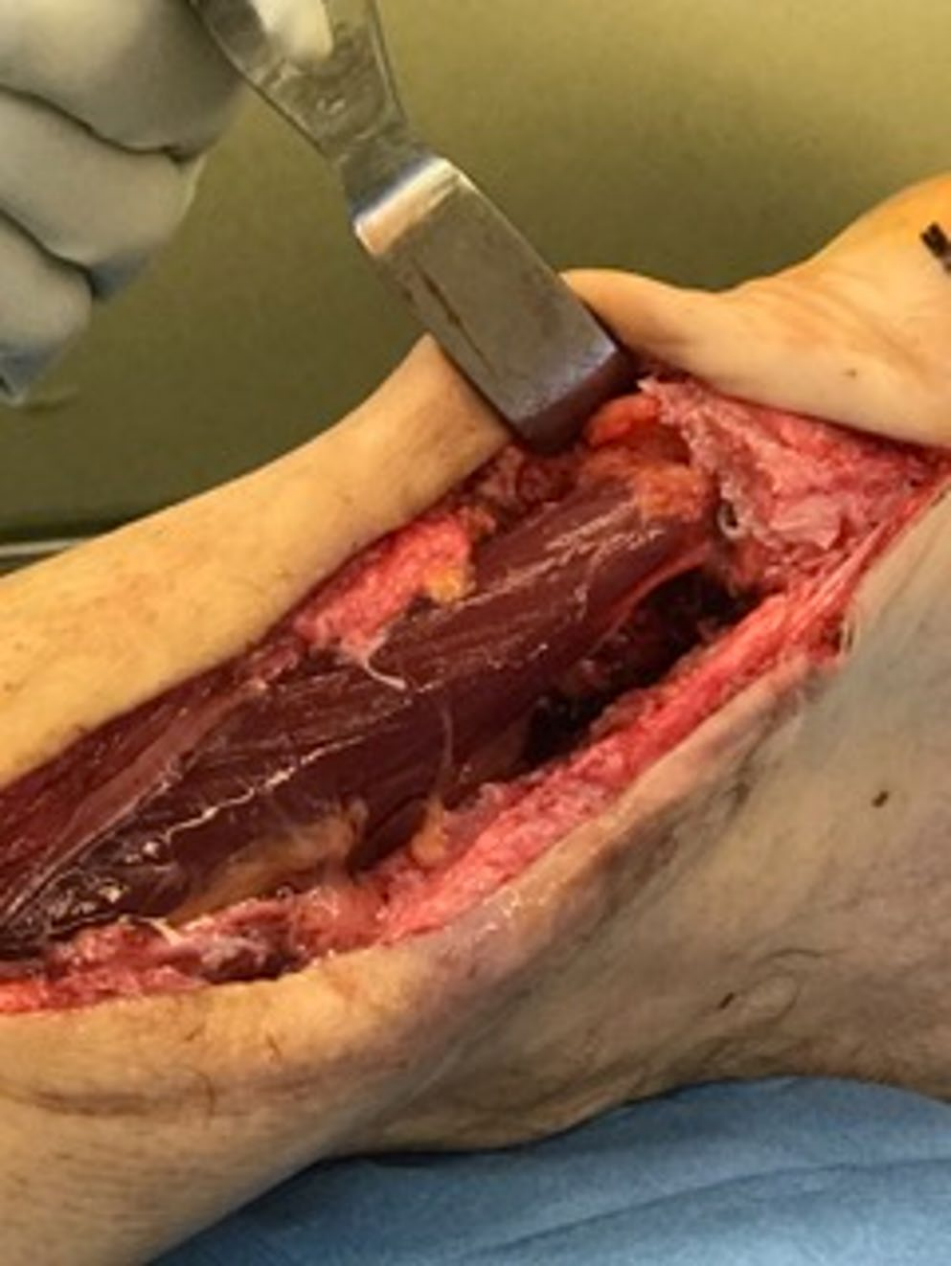
Intra-operative photo of example FHL tendon transfer. Author currently does procedure with smaller incision (not pictured). A posterior incision is made just medial to midline, paratenon split and deep fascia overlying FHL released. Careful dissection is done following the FHL tendon distally to posteromedial talar process. FHL is then transected as distally as possible, a nonabsorbable whipstitch is placed, and the tendon diameter is sized. A calcaneal tunnel is drilled with Beath needle starting just anterior to achilles in central portion of posterior tuberosity, aiming plantar and distal to create a long vertical oblique tunnel, and avoiding penetration through inferior calcaneal cortex. After overdrilling with reamer matching FHL diameter, whipstitch suture ends are passed through the eyelet of the Beath needle and pulled out the plantar heel as needle is removed. With the ankle held in 20 degrees of plantarflexion, the FHL is dunked into the tunnel, tensioned tight via pulling inferiorly on whipstitches, and fixated with an interference screw

The most often used and preferred tendon transfer for secondary Achilles repair, including chronic ruptures and revisions, is the flexor hallucis longus (FHL) [12]. Peroneus brevis (PB) is an alternative option [19]. FHL serves as an ideal donor tendon due to its accessibility just posterior to the Achilles, length of the tendon transfer, biomechanical positioning in line with the Achilles, in-phase transfer, and larger muscle cross sectional area resulting in greater strength [12, 19] The FHL may be either tenodesed to the calcaneus via passage through a vertical oblique tunnel with interference screw fixation **(**Fig. 3**)** or directly sutured to the distal Achilles stump plus or minus a transverse calcaneal tuberosity tunnel.

### Secondary Reconstruction With Allograft

In reconstruction of chronic Achilles tendon ruptures without the use of tendon transfers, several allograft options exist. Frozen Achilles tendon allograft is readily available from tissue banks and may be used as a substitute for large defects. Kraeutler et al. describe their technique in a recent review [20]. A recent study involving 8 patients who underwent secondary repair using allograft compared to 9 patients who underwent autologous techniques [21]. There was no significant difference in AOFAS or ATRS scores, and 100% of patients were able to achieve a single leg heel rise. Overall, studies of allograft Achilles repair are limited by small sample size, and further research is needed to determine the utility of this technique compared to other secondary repair techniques [22, 23].

## Functional Outcomes After Achilles Treatment

Functional outcomes are assessed by both objective measures including strength, range of motion (ROM), performance tests and the limb symmetry index (LSI) as well as subjective measures including patient reported outcomes (PROs). Popular PROs include the Achilles tendon Rupture Score (ATRS), American Orthopaedic Foot & Ankle Society (AOFAS) ankle-hindfoot scores and Patient-Reported Outcomes Measurement Information System (PROMIS). Functional outcomes vary by treatment modality and surgical technique.

Nonsurgical treatment with an early mobilization protocol has demonstrated comparable functional outcomes to surgical treatment [24]. Furthermore, the UKSTAR trial showed no significant difference in ATRS scores between those treated with early mobilization in a functional brace and traditional plaster casting [25]. There is no increased benefit with adjuncts such as PRP in terms of pain, goal attainment or PROs [26]. There is conflicting evidence regarding long term functional differences between nonsurgical and surgical management. However, surgery results in earlier restoration of up to 18% more strength over the entire range of motion [27]. Thus, studies have shown a significantly higher rate of return to sport in athletes of all ages treated surgically when compared to those treated conservatively [28]. As a result, young athletes are rarely treated non-surgically.

In patients treated surgically, there is conflicting evidence regarding whether the type of surgical repair influences functional outcomes. Surgical treatment may involve conventional open, minimally invasive surgery (MIS), endoscopic and hybrid techniques. MIS techniques include both suture bridge constructs such as the Percutaneous Achilles Repair System (PARS; Arthrex, Inc., Naples, FL) or Achillion (Integra, Plainsboro, NJ, US) and artificial ligament augmentation constructs such as the Ligament augment reconstruction system (LARS). The PARS is commonly used for acute ruptures, whereas the LARS is reserved for chronic ruptures or tendinopathic cases. MIS techniques have been shown to have early functional advantages including decreased pain, improved early ROM, earlier return to work and superior PRO scores without sustained long-term benefits [29–33]. When comparing MIS techniques and MIS and endoscopic techniques, multiple studies found no significant difference in ATRS or PROMIS scores long term [30–32].

Additional preoperative, intra-operative and postoperative factors may influence functional outcomes. Preoperative depression, age greater than 50 years and delay of surgery greater than 14 days have been shown to result in inferior functional outcomes [34–36] Quality and tightness of repair has been associated with increased heel rise height index at 1-year [37]. Postoperatively, early weightbearing with MIS techniques was associated with faster return to work and higher ATRS scores [33]. This study also demonstrated that open repair with late weightbearing demonstrated improved heel-raise and early ATRS scores.

### Gender Differences in Achilles Tendon Ruptures

ATR has been previously viewed as an injury predominantly affecting middle-aged males, with incidence ratios (male: female) reported between 5:1 and 7:1 [38]. Recent epidemiological data suggests that ATRs are becoming increasingly common in women, driven largely by rising female participation in high-impact plyometric sports. Regarding clinical outcomes, female sex may be a predictor of worse functional recovery [39]. In a cohort study of 856 patients by Larsson et al., women scored an average of 7.8 points lower on the ATRS compared to male counterparts [39]. Functional objective measures have also been shown to demonstrate sex disparities. Larsson et al. also found significantly larger deficits in heel-rise height and heel-rise work LSI in women patients compared to men at the three month post-operative mark [39]. With operative treatment, Silbernagel et al. also found higher rates of complications in female patients, including a greater risk of wound complications [40]. Further research must be done to include reporting of and separate analysis of gender-based data, as previous work has shown potential recovery disparities.

## Postoperative Rehabilitation Protocols

### Primary Repair

An example rehabilitation protocol used by our institution is included in Appendix A. There is good evidence to demonstrate that early weight bearing and ROM is safe in primary Achilles repair. Two randomized controlled trials evaluated early rehabilitation after primary Achilles tendon repair compared to nonoperative treatment with early functional rehabilitation [41, 42]. They found that early rehabilitation in the surgical group did not increase risk of rerupture or impact functional outcome measures. Smaller studies have also investigated this. Hrnack et al. evaluated outcomes with accelerated rehabilitation (weight bearing 7 days postoperatively, active ROM at 6 weeks, and concentric/eccentric exercises at 12 weeks) after open primary repair [43]. In their series of 15 patients, they had excellent AOFAS scores and 100% of patients were able to perform a single leg heel rise. Return to sports was achieved at 4 to 6 months. A larger study involving 64 patients undergoing open repair evaluated early mobilization, defined as active ROM beginning immediately [44]. Patients were initially immobilized part time in a brace and were weaned from the brace at 4–6 weeks. At 100 days, all patients had resumed their normal daily activities. Functional outcome scores were not obtained.

Additionally, blood flow restriction (BFR) therapy has recently been utilized for early post-surgical rehabilitation following Achilles ruptures. BFR is measured using a specialized cuff to determine a patient’s limb occlusion pressure, allowing therapy to be safely individualized to a specific percentage of their full arterial blockage [45]. This technique provides benefits by stimulating muscle hypertrophy and increasing strength at significantly lower exercise loads, preventing atrophy while protecting healing tissues. In a randomized controlled trial following Achilles tendon ruptures, Hansen et al. demonstrated that implementing BFR during rehabilitation resulted in significantly greater ankle plantarflexion and absolute calf strength at three months post-surgery compared to conventional physical therapy protocols [46]. Further information regarding recovery guidelines is provided below, as Table 1 compares phases of rehabilitation after operative and non-operative management.

**Table 1:** Phases of Recovery in Non-Operative and Operative Management for Achilles Tendon Ruptures

Phase	Timeline	Non-Operative Highlights	Operative Highlights
I: Protection	0–2 Weeks	Strict NWB; focus on proximal hip/knee strength.	NWB in splint/boot; gentle AROM (no DF); submaximal isometrics.
II: Early Loading	2–6 Weeks	PWB in boot with heel lifts; seated heel raises within limited range.	WBAT in boot with heel lifts weaned by week 5; initiation of BFR training and stationary bike (in boot).
III: Progressive Loading	6–12 Weeks	Wean boot/lifts (weeks 8–10); normalize gait; bilateral heel raises.	Wean boot; progress to single-leg heel raise from flat ground by week 12.
IV: Advanced Loading	12–24 Weeks	Weighted calf loading; initiate jogging intervals if 80% LSI met. High risk of rerupture.	Weighted calf loading; initiate plyometrics and running protocols.
V: Return to Sport	6 + Months	Sport-specific agility; targeting 90–100% LSI strength.	High-impact training; clearance via Return to Sport Protocol (Table 2).

*NWB = non-weight bearing; *AROM* = active range of motion; *DF* = dorsiflexion; *PWB* = partial weight bearing; *WBAT* = weight bearing as tolerated; *BFR* = Blood Flow restriction; *LSI* = Limb Symmetry Index

### Tendon Transfers and Secondary Repair

Data is limited regarding postoperative rehabilitation protocols after tendon transfers and secondary repair. In general, immobilization is encouraged for longer periods of time than for primary repair to protect the more tenuous repair construct. One study from Switzerland retrospectively reviewed 28 patients who had undergone FHL transfer with transosseous tenodesis [47]. Patients were immobilized for 3 weeks in plantarflexion before beginning partial weight bearing. Full weight bearing was allowed at 4 weeks, and all heel wedges were removed during the 10th week. Full return to sport was allowed at 9 months. A retrospective review of 9 patients who underwent dual semitendinosus allograft reconstruction described their post-op protocol to include 2 weeks of splint immobilization with transition into boot, followed by progressive weightbearing and ankle range of motion starting at 6 weeks after surgery [48]. Similarly, a review of 8 patients who underwent allograft reconstruction required 3 weeks of cast immobilization [49]. Passive plantarflexion was allowed at 3 weeks in an articulating boot, and progressive weight bearing was delayed until 8 weeks postoperatively. In the authors’ treatment paradigm, due to use of interference screw fixation of the FHL tendon transfer which provides excellent tendon fixation in calcaneus, these reconstructions are rehabilitated similarly to primary repairs with no adverse events observed.

### Return to Sport (RTS) Criteria

The decision to clear an athlete for RTS following an Achilles tendon rupture (ATR) has historically relied on time-based protocols. Current best practices, however, mandate a shift towards objective based milestones to mitigate reinjury [50, 51]. Readiness for RTS requires a multidisciplinary assessment. In the clinic, surgeons must ensure resolution of baseline physical impairments, while physical therapists (PTs) quantify muscular endurance, strength recovery, and psychological readiness [52]. When isolating the gastrocnemius-soleus complex, the single leg heel rise (SLHR) test remains the clinical gold standard. Athletes should achieve a Limb Symmetry Index (LSI) of at least 85–90% in total work before full athletic clearance may be considered [51, 53]. Furthermore, an on-field rehabilitation protocol by Buckthorpe et al. determined four criteria aimed at RTS [54]. These included restoring movement quality, physical conditioning, restoring sport specific skill, and progressive development of chronic training load [54]. A summary of criteria are summarized below in Table 2 and Appendix A [51–53, 55].

**Table 2:** Return to Sport Criteria for Treating Physicians and Physical Therapists for Achilles Ruptures

Treating Physician Criteria	Physical Therapy Criteria
Pain free palpation of the Achilles tendon	Single-Leg Heel Rise (SLHR) LSI > 85–90%
Full and symmetrical ankle dorsiflexion and plantarflexion	Isokinetic plantar flexion peak torque LSI > 90%
Absence of reactive effusion after activity	High psychological readiness
Normal gait mechanics on level and uneven surfaces	95% Symmetry in Calf Circumference at 10 cm distal to tibial tubercle

As ATR impacts the tendon’s cycle of stretching and shortening, especially tested in dynamic movements, closed chain strength tests alone are insufficient for athlete RTS criteria [56]. Functional performance tests (FPTs) are essential for evaluating reactive strength, neuromuscular control, and power symmetry [57]. Four FPTs are summarized below in Table 3.

**Table 3:** Functional Performance Tests in ATR Recovery

Functional Performance Test	Primary Variable Assessed	RTS Target
Single-Leg Hop for Distance	Forward explosive power and landing mechanics	LSI > 90%
Drop Jump	Reactive strength index and tendon stiffness	LSI > 90%; Contact time < 0.25s
Y Balance Test	Lateral and forward power and dynamic ankle stabilization	LSI > 90%
Agility T-Test	Multidirectional cutting and rapid deceleration	Within 10% of baseline prior to injury

Importantly, recognizing that biomechanical compensation can overload the contralateral tendon is crucial. A randomized controlled trial done by Willits et al. found measurable asymmetries in isokinetic testing between operated and contralateral Achilles tendons [57]. These discrepancies, however, fail to translate into meaningful discrepancies in RTS [58]. Patient reported outcome measures, such as the ATRS, improve steadily over time in operative and non-operative management for recovering tendons [59]. Still, continuing isolated calf strengthening and plyometric maintenance even after RTS may provide bilateral tendon protection over time due to the isokinetic differences seen [58].

## Conclusion

The management of Achilles tendon injuries remains a complex clinical challenge that demands a comprehensive understanding of the tendon’s fundamental biomechanics and distinct pathophysiological differences between acute and chronic presentations. Selection between operative and non-operative management requires individualized consideration, and adhering to evidence-based rehabilitation protocols, such as the one provided, is paramount to positive functional outcome. Navigating these patient specific treatment options for various patient populations promotes optimal recovery in the short and long term.

## Key References

- B MS, F BE, M ATK, Karin R, Madeleine A, Wolfram G, Faisal B, Morten V, Svend U, E HS (2022) Nonoperative or Surgical Treatment of Acute Achilles’ Tendon Rupture. New England Journal of Medicine 386: 1409-1420.
  - ○ Multicenter randomized controlled trial which compared nonoperative treatment, open repair and minimally invasive surgery in adults with achilles rupture, finding no difference in functional outcomes between treatment groups but surgical treatment was protective against rerupture.
- Irfan SA, Ahmed S, Ashkar A, Heyes G, Khan MW, Ahsan Nawaz SM, Siddiqui AA, Mustafa H (2025) Comparative effectiveness of weight-bearing strategies on functional recovery in acute Achilles tendon rupture: A network meta-analysis. Foot and Ankle Surgery 31:561–569
  - ○ Meta-analysis performed which included randomized controlled trials and evaluated the impact of early weightbearing on functional outcomes after achilles rupture, with minimal invasive repair plus late weightbearing (after 2 weeks post-operatively) group having lowest rerupture rate and minimal invasive repair plus early weightbearing with quicker work return and higher late outcome scores.
- Cao Y, Li X, Cui Z, Lv Y, Si G (2025) Open surgery and minimally invasive repair of acute Achilles tendon rupture: stratified outcomes based on immobilization duration in a prospective cohort study. J Orthop Surg Res. https://doi.org/10.1186/S13018-025-06019-0
  - ○ Prospective study of 474 patients which compared open surgery to modern repair technique (minimally invasive surgery) and length of immobilization after surgery, finding similar outcomes but faster early recovery in MIS group.
- Larson E, Brorsson A, Carling M, Johansson C, Carmont MR, Nilsson Helander K (2022). Sex differences in patients’ recovery following an acute Achilles tendon rupture- a large cohort study. BMC Musculoskelet Disord. https://doi.org/10.119=86/s1289`-011-05875-9
  - ○ A first large cohort study which focused on evaluating gender differences after achilles tendon rupture repair, which are significant between males and females with respect to outcome and objective functional measures.
- Marrone W, Andrews R, Reynolds A, Vignona P, Patel S, O’malley M (2024) Rehabilitation and Return to Sports after Achilles Tendon Repair. Int J Sports Phys Ther 19:1152–1165.
  - ○ Excellent review article on optimizing rehabilitation for the athlete after achilles rupture, including use of newer techniques such as Blood Flow Restriction Therapy (BFR) and return to sport guidelines.

## Supplementary Information

Below is the link to the electronic supplementary material.

Supplementary Material 1 (PDF 281 KB)

## Data Availability

No datasets were generated or analysed during the current study.
