# Supplementary material for: Parsing Through the Data on Achilles Tendon Rupture Management, Rehab and Sports Return Criteria: a Current Literature Review
Source: Curr Rev Musculoskelet Med. 2026 Jul 1;19(1):55. doi: 10.1007/s12178-026-10043-w (PMC13319554; doi:10.1007/s12178-026-10043-w)
Supplement: Supplementary file 1 — Supplementary Material 1 (PDF 281 KB) [file 12178_2026_10043_MOESM1_ESM.pdf]

University Hospitals Rehabilitation Services

University Hospitals Orthopedics

# Achilles Repair Guidelines

An Achilles tendon rupture is a common and often debilitating injury that typically occurs during activities that involve sudden acceleration or changes in direction, such as jumping, sprinting or pushing off forcefully. It most frequently affects men between the ages of 30 and 50 and often results from a sudden overload of the tendon, particularly in the presence of degenerative changes, an inadequate warm-up, or a history of pain or tendinopathy.

Surgical repair is often recommended for active individuals or those with complete ruptures to restore tendon continuity, optimize strength recovery and reduce the risk of re-rupture. The procedure involves approximating the torn ends of the tendon using sutures, either through a traditional open technique or a minimally invasive (percutaneous) approach. The choice of surgical technique depends on factors such as the tear's location and extent, the patient's activity level and surgeon preference.

Following repair, recovery is expected to take 6-9 months, with a return to sport potentially taking 9-12 months, depending on the injury's severity and the specific sport's demands. Rehabilitation plays a critical role in guiding tendon healing while gradually restoring mobility, strength and function. These clinical guidelines provide a structured, phase-based rehabilitation framework to ensure a safe and effective recovery tailored to the surgical technique and individual patient progress. **Progression is both time and criterion-based, dependent on soft tissue healing and clinical evaluation.**

Listed below is a suggested Achilles Tendon Repair rehabilitation guideline. Please confirm with the surgeon for specific precautions and guidelines if necessary.

Considerations Following Achilles Tendon Repair

- Type of repair, percutaneous vs open, may alter healing time frames and guideline progression in the early phases; open repairs often used for revision and tendon transfer/augmentation surgeries
- Emphasis placed on wound healing and would closure – minimize friction over incisions, inspect wound as able, alert physician immediately if delayed closure is suspected
- Current literature favors early weight-bearing (WB) – protected WB in boot with assistive device 2-4 weeks post-op is recommended
- Early mobilization of ankle is favored – immediate free plantarflexion of the ankle is recommended
- Begin progressive loading from early stages post-operatively to aid in restoration of mechanical and elastic properties of tendon
- Use of neuromuscular electrical stimulation (NMES) to minimize atrophy to gastrocnemius musculature
- Early use of blood flow restriction (BFR) between 2-4 weeks on surgical limb to help promote overall lower extremity strengthening is recommended
- Facilitate calf musculature activation – if available, use biofeedback for neuromuscular education
- Aquatic therapy – if available, can be utilized to reinforce movement quality with single leg strength and tendon stretch-shortening capabilities
- Compensatory patterns are easily formed in mid to late stages with single calf raise
- Exercise list is not exhaustive for each stage – use creativity and clinical judgement

# General Information and Timeframes

**Precautions**
- No dorsiflexion range of motion (ROM) past neutral for 8 weeks to minimize tendon elongation – greatest elongation occurs between 2- and 6-weeks post-op
- Symmetrical ankle ROM 8-10 weeks
- Full weight-bearing heel raises 8-10 weeks
- Avoid calf stretching for 12-16 weeks or longer to prevent tendon elongation
- Return to plyometrics and running 12-16 weeks
- Return to sport progression and testing 24 weeks

### ROM/Manual Therapy
- Active range of motion (AROM)/mobilization used to restore normal joint ROM by 10-12 weeks post-op, see individual phases for ROM restrictions

### Effusion
- Manage swelling and edema with cryotherapy, elevation, compression and soft tissue mobilization

### Therapeutic Exercise
- Progress non-weight bearing to full weight-bearing exercises as described in individual phases

### Functional Patient Outcomes
- FAAM (Foot and Ankle Ability Measure), Activities of Daily Living (ADL) and Sport Subscale
- Patient-Specific Functional Scale (PSFS)

### Considerations Regarding Running and Plyometrics
- Symmetrical ankle dorsiflexion and plantarflexion ROM
- 80% Limb Symmetry Index (LSI) plantarflexion strength with isometric testing at 0 and 20 degrees of plantarflexion
- 80% LSI single leg heel raise test through full ROM
- 1.5x body weight with isometric seated plantarflexion strength
- Good neuromuscular control and mechanics with single leg squatting
- Normalized gait and jogging mechanics
- Initiate between weeks 12-16
- See Appendix C for Phased Running Progression

### Criteria for Discharge (Athletic)
- Symmetrical ankle dorsiflexion and plantarflexion ROM
- Symmetrical dorsiflexion lunge test
- 95% symmetry calf circumference at 10cm distal to the tibial tubercle
- 90% LSI plantarflexion strength with isometric testing at 0 and 20 degrees plantarflexion
- 1.5-2.0x body weight with isometric seated plantarflexion strength
- 90% LSI single heel raise test through full ROM
- 90% LSI on lower extremity (LE) Y-balance with proper LE mechanics
- 90% LSI on single leg hop testing
- Expected time frame between 9-12 months
- See Appendix D for return to sport testing

### Criteria for Discharge (Non-Athletic)
- Symmetrical ankle dorsiflexion and plantar flexion ROM
- Symmetrical dorsiflexion lunge test
- 80-90% LSI plantarflexion strength isometric testing
- 80-90% LSI single leg heel raise test through full ROM
- Normalized gait mechanics

# General Information and Timeframes

**Achilles Tendon Repair Yellow Flags and Red Flags**

YELLOW FLAGS

- Unhelpful beliefs about pain or expectations of poor outcomes
- Catastrophizing or fear of movement
- Over reliance on passive treatments

RED FLAGS

- Signs of deep vein thrombosis (DVT) - refer directly to emergency department:
  - Localized tenderness along the distribution of deep venous system
  - Swelling of entire LE or calf swelling >3 cm compared to asymptomatic limb
  - Pitting edema
  - Erythema
  - Collateral superficial veins

- Signs of infection (contact surgeon):
  - Fever, chills, night sweats, redness, warmth around wound or incision
  - Abnormal pain at surgical site
  - Drainage
  - Sudden loss of range of motion or inability to bear weight

# Phase I Protection

**PHASE I: 0 day - 2 weeks**

**Precautions**

- Non-weight bearing with crutches or walker for 2 weeks unless otherwise directed by the surgeon
- Immobilization in a posterior splint or controlled ankle motion (CAM) boot set in plantarflexion (~20 degrees)
- No active dorsiflexion past neutral, no passive dorsiflexion

**Goals**

- Protect surgical repair
- Control pain and swelling
- Ensure closure of incision
- Prevent complication (DVT, stiffness)
- Begin safe ROM
- Maintain proximal muscle strength

**Interventions** *(Exercise list is not exhaustive)*

**Therapeutic Exercise / Activities / NMRE**

- Proximal hip and LE strengthening: 3-way straight leg raise (SLR), long arc quad (LAQ), HSC
- Intrinsic foot (towel crunch, arch doming, toe yoga)
- Gentle AROM (circles, inversion/eversion), no dorsiflexion
- Submaximal isometrics in all ankle planes including plantarflexion in boot
- Blood flow restriction training on nonsurgical limb for lower extremity musculature

**Manual Therapy**

- Joint mobilizations: tibiofibular, subtalar, mid foot, forefoot as needed (as allowable by removable splint or CAM boot)
- PROM plantarflexion as tolerated

**Range of Motion**

- No passive or active dorsiflexion
- Gentle AROM (circles, inversion/eversion), no dorsiflexion

**Modalities**

- Ice, elevation and compression

**Gait**

- Non-weight bearing with crutches unless directed otherwise by surgeon

**Criteria to Progress to Phase II**

*Criteria supersedes time for progression to next phase

1. Adequate pain and swelling control
2. Good wound closure
3. Able to tolerate PROM of ankle (no dorsiflexion) and regional foot mobilization
4. No signs of infection of DVT

**Appendix A**

**Appendix A: Weight-bearing Progression**

- Non-weight bearing in post-op splint or cast x 2 weeks
- Partial weight-bearing in clinic to begin in boot with heel lifts weeks 2-4 per surgeon
- WBAT in boot with heel lifts at 4 weeks
- Discharge assistive device when weight-bearing is pain free with improving gait mechanics (4-6 weeks)
- Wean 1st heel lift weeks 5-6, 2nd weeks 6-7
  *Criteria to wean is pain free ambulation and dorsiflexion to neutral
- Transition to shoe approx. 8-10 weeks, goal is flat shoe ambulation at 10 weeks
  *May use 1-2 heel lifts in shoe and wean weekly
- Discharge boot criteria: no pain, active dorsiflexion to neutral, good gait mechanics

# Phase II Early Loading Phase

**PHASE II: 2 - 6 weeks**

**Precautions**

- No passive stretching into dorsiflexion to prevent tendon elongation
- No active dorsiflexion past neutral
- Weight bearing in boot with wedges as directed by surgeon
- Avoid walking out of boot

**Goals**

- Control pain and swelling
- Gradually increase to full weight-bearing in boot
- Prevent muscle atrophy
- Maintain mobility of adjacent joints
- Initiate ankle strengthening

**Interventions** *(Exercise list is not exhaustive)*

**Therapeutic Exercise**

- Continue AROM in pain free ranges (circles, alphabet)
- Continue submaximal pain-free isometrics
- Plantarflexion isometrics in terminal plantarflexion
- Initiate partial weight-bearing proprioception (weight shifts, BAPs board)
- Lower extremity strengthening emphasizing quads and hips utilizing BFR when appropriate
- Stationary bike (in boot initially)
- 3-way TheraBand (within ROM restrictions)
- Seated heel raises

**Modalities**

- Continue modalities to control swelling/inflammation
- Initiate blood flow restriction training weeks 2-4 on surgical limb and contralateral limb

**Manual**

- Ankle PROM (no dorsiflexion)
- Talocrural joint mobilizations (in neutral or plantarflexion)
- Soft tissue mobilization to calf and tendon (once incision is closed)

**Gait**

- Weight-bearing in boot with heel wedges as instructed by surgeon (typical progression to full weight-bearing at 4-6 weeks)

**Criteria to Progress to Phase III** *Criteria supersedes time for progression to next phase

1. Good tolerance to weight-bearing progression in boot with assistive device
   *DC device when non-painful gait achieved in boot
2. Active ankle dorsiflexion to neutral
3. Good tolerance to submaximal ankle isometrics

# Phase III Progressive Loading

**PHASE III: 6 - 12 weeks**

**Precautions**

- No passive gastrocnemius stretching for 12 weeks – only perform if necessary
- Avoid running/jumping activities
- Limit dorsiflexion to neutral for 8 weeks to avoid tendon elongation

**Goals**

- Progressive weight-bearing, wean heel lifts from boot → shoes with heel lift → shoe with no lift
- Progressive AROM to tolerance, normalizing ankle plantarflexion, inversion and eversion. Caution with dorsiflexion
- Good tolerance to gradual progression of plantarflexion loading
- Normalize gait pattern and maximize functional mobility out of boot and without an assistive device

**Interventions** *(Exercise list is not exhaustive)*

**Range of Motion**

- Continue with open chain ankle AROM in all major planes: ankle circles, ankle alphabets, ankle/inversion/eversion (minimizing ankle dorsiflexion past neutral until 8 weeks)
- Seated biomechanical ankle platform system (BAPS) board/Rock board (circles, inversion/eversion, avoiding ankle dorsiflexion past neutral until week 8)
- Weeks 10-12: utilizing supported squatting/lunging to promote increasing closed chain ankle dorsiflexion (modify range to tolerance)

**Therapeutic Exercises**

- Continue ankle strengthening and advance proprioception activities gradually from previous phase
- Seated heel raise from flat ground
- Partial weight-bearing calf raises on shuttle, progress to flat ground heel raises starting week 8/9, single leg by week 12
- Progress proximal lower extremity closed chain and open chain strengthening (squats, steps, lunges)
- Heel taps within available ROM
- Stationary bike
- Use of antigravity treadmill if available to restore normal gait mechanics

**Late exercises**

- Progressing to weight-bearing bilateral heel raises, gradual progression to single leg heel raise from flat ground at end of phase
- Progression of load with proximal lower extremity strengthening (squats, lunges, step ups)
- Treadmill ambulation once in shoe (gait retraining, aerobic endurance)
- Progression to dynamic balance exercises: stable surface → unstable surface/perturbations

**Balance progressions**

- Initiate balance exercises on unstable surfaces

# Phase III Progressive Loading

**Interventions** *(Exercise list is not exhaustive)*

**Gait Training**

- 6-8 weeks pre-gait training as patient transitions to shoe (static and dynamic weight shifting, unilateral marching, step throughs with assistive device → without assistive device
- Criteria to discharge boot: no pain, active dorsiflexion to neutral, normalizing gait mechanics

**Manual**

- Joint mobilizations as needed to normalize joint mechanics
- Avoid stretching of the gastrocnemius

**Modalities**

- As needed to control swelling/soreness

**Aquatics (if available)**

- Exercises focused on restoring proper gait mechanics and calf strengthening

**Criteria to Progress to Phase IV** *Criteria supersedes time for progression to next phase

1. Discharge of assistive device
2. Transition to athletic shoes with or without lift
3. Able to complete single leg heel raise from flat ground with minimal discomfort
4. Full ROM of involved foot/ankle (calf tightness expected)

# Phase IV Advanced Loading Phase

**PHASE IV: 12 - 24 weeks**

**Precautions**

- Only stretch passively or statically into dorsiflexion if deficits in range of motion are observed
- No running or plyometrics until criteria is met

**Goals**

- Early-phase ankle plantarflexion strength at least 80% LSI compared to uninvolved limb, late phase 90% for return to sport training
- Normalizing ankle AROM and passive range of motion (PROM) in all planes
- Improving tolerance to advanced loading (eccentric, plyometric, multi-planer stability challenges)
- Progressively return to recreational activities by increasing aerobic participation and restoring cardiovascular fitness

**Interventions** *(Exercise list is not exhaustive)*

**Therapeutic Exercises**

- Progress calf loading in weight-bearing position (flat ground → weighted flat ground v from wedge)
- Progress static and dynamic balance training
  (static → unstable surfaces → perturbations → dynamic stabilization)
- Progress loaded compound lower extremity lifts (squats, lunges, step-up)
- Introduction of beginning plyometric program
  Criteria: symmetrical ankle ROM, 80% LSI plantarflexion strength
- Introduce return to running when appropriate
  Criteria: symmetrical ankle ROM, 80% LSI plantarflexion strength
- Initiate walk/jog intervals and progress as tolerated (see Appendix C)
- Utilize antigravity treadmill if available

**Manual**

- Address ankle mobility restrictions as needed

**Criteria to Progress to Phase V** *Physician clearance required

1. Good tolerance with return to running and plyometric program
2. Symmetrical ankle dorsiflexion ROM
3. 80% LSI plantarflexion strength (single leg heel raise to failure test, isometric testing in 0-20 degrees plantarflexion)

# Phase V Return to Sport/Activity

**PHASE V: 6+ months**

**Precautions and Considerations for Return to Recreational Activities**

*This phase is only required for patients who wish to participate in moderate-impact recreational sports and/or return to work that involve walking on uneven ground, climbing and carrying objects like construction or other trades.*

- Patients who wish to return to moderate-impact activities such as doubles tennis, doubles pickle ball, horseback riding or downhill skiing should only be on a case-by-case situation and should be cleared by their surgeon, meet the criteria and complete moderate-impact training

**Goals**

- Clearance of Return to Sport Protocol (see appendix)
- Clearance from physician for return to sport
- Good tolerance to all return to sport-based activities

**Interventions** *(Exercise list is not exhaustive)*

**Therapeutic Exercises**

- Agility based training (running/hopping/cutting)
- Return to sports-specific activities

**Criteria to progress to moderate impact activities***

*Physician clearance required

1. Satisfactory completion of rehab program
2. Achievement of pre-testing criteria (see appendix)
3. Passing of RTS protocol
4. Clearance from physician

## Appendix C: Phased Running Progression

Name:
*If post-op, patient has met specific criteria to begin running program:

### Dynamic Warm up

5) Double Leg Squats x 10 reps
5) Single Leg Squats x 5-10 reps
5) Single Leg Heel Rise x 10 reps (each side)
5) Lateral Band walks x 10 reps (each side)
5) Plank x 30 seconds (each side)

### Phase I: Walking

- Patient able to walk 30 minutes at 3.5 mph without pain

### Phase II: Plyometrics

- Ladders: Forward, lateral, single leg, in/out, zigzag, rest 2 minutes, repeat 3x
- Double leg line jumps: Front and Back 3 x 12 reps
- Double leg line jumps: Medial and Lateral 3 x 12 reps
- Alternating step and hold forward: 3 x 10 each limb
- Alternating single leg hops with bounce: 3 x 10 each limb

*Goal: To work your way up to 500-600 foot contacts with completion of phase II before progressing to phase III

### Phase III: Walk/Jog program

- Recommend starting on treadmill to control speed and distance
- Pace: Comfortable jog where you can hold a conversation without being out of breath
- Run every other day for first two weeks, at least two days of running on each level before progressing
- Tips: Avoid hills/inclines initially, no speed work, work on form

| Walk Interval | Run Interval | Repetitions | Days |
|---|---|---|---|
| 4 minutes | 1 minute | 3-6 | 2-3 |
| 3 minutes | 2 minutes | 3-6 | 2-3 |
| 2 minutes | 3 minutes | 3-6 | 2-3 |
| 1 minute | 4 minutes | 3-6 | 2-3 |
| 0 | 30 minutes | 1 | 3 |

*Instructions*

- The runner is to take at least one day off in between running days
- The runner can progress to the next phase once they are able to complete 6 reps of the run time without increased pain or swelling
- Do not progress to the next phase if one of the following occurs:
  - o Sharp pain during run
  - o Pain that worsens as patient continues running
  - o Pain is so severe that it causes patient to alter gait
- After completion of final phase, increase weekly mileage by 10-30% or initiate return to sprinting progression
- Slight stiffness at beginning of run, resolves within 10 minutes

**Acceptable:** Continue to progress training

- General muscle soreness
- Slight joint discomfort after workout or next day that resolves in 24 hours
- Slight stiffness at beginning of run, re-solves within 10 minutes

**Unacceptable:** Back off training

- Pain that lasts longer than 24 hours after workout
- Pain that is present at beginning of run and becomes worse as run continues, and changes gait
- Pain keeping patient awake at night

*This Clinical Guideline may need to be modified to meet the needs of a specific patient.
The model should not replace clinical judgment.

## Appendix D: Return to Sport Testing

Name:______________________________ Date of Test:___________________ Months Post-op:____________
MR#:______________________________ Procedure:________________________________

**Satisfactory Clinical Examination**
1. Appropriate time from injury/surgery for healing
2. Completed rehabilitation program—Understands HEP
3. Pain free full ankle ROM
4. 95% symmetry calf circumference at 10cm distal to the tibial tubercle
5. Completion of running program and plyometrics with 0/10 pain
6. No kinesiophobia

**FAAM ADLs Subscale:** ___________
* ≥ 100% for RTS

**FAAM Sports Subscale:** _____________
* ≥ 90% for practice, ≥ 100% for RTS

**ALR-RSI Questionnaire:** _________
* ≥ 90 for RTS

**Single Heel Raise Test** Right:_________ Left:_________ LSI:_________ **Pass:** ☐ **YES** ☐ **NO**
* LSI ≥90% to pass, *≥ 25-30 reps to pass

**Dorsiflexion Lunge Test** Right:_________ Left:_________ LSI:_________ **Pass:** ☐ **YES** ☐ **NO**
* LSI ≥90% to pass

**Strength Testing (HHD or Force Frame)** **Pass:** ☐ **YES** ☐ **NO**

| | Left (lbs.) | Right (lbs.) | LSI |
|---|---|---|---|
| Plantarflexion | / / | / / | |

* LSI ≥90% to pass *1.5-2.0x BW to pass

**LE Y-Balance Test** **Pass:** ☐ **YES** ☐ **NO**

| | Left | Right | Difference* | Limb Length: (ASIS to med mal) | |
|---|---|---|---|---|---|
| Anterior | / / | / / | | Left | Right |
| Posteromedial | / / | / / | | | |
| Posterolateral | / / | / / | | | |
| Composite Score^ | | | | | |

3 trials, record maximal reach in each direction
*Difference should be less than 4cm for return to sport; <4 cm = pass
^Composite Score= (Medial + posteromedial + posterolateral)/(3x limb length) X 100

University Hospitals Rehabilitation Services

University Hospitals Orthopedics

## Appendix D: Return to Sport Testing

Name:______________________________ Date of Test:__________________ Months Post-op:____________

MR#:______________________________ Procedure:______________________________

**Functional Hop Testing** **Pass:** ☐ **YES** ☐ **NO**

| | Uninvolved Side | | | | Involved Side | | | | LSI |
|---|---|---|---|---|---|---|---|---|---|
| Plantarflexion | 1 | 2 | 3 | Avg | 1 | 2 | 3 | Avg | |
| | | | | | | | | | |
| Triple Hop (cm) | 1 | 2 | 3 | Avg | 1 | 2 | 3 | Avg | |
| | | | | | | | | | |
| Cross Over Triple Hop (cm) | 1 | 2 | 3 | Avg | 1 | 2 | 3 | Avg | |
| | | | | | | | | | |

*≥ 90% for RTS

**T Agility Drill**

| Trial 1/2/3 | Best |
|---|---|
| / / | |

*<11 seconds to pass

**Able to complete sport-specific drills in clinic with good motor control/movement patterns (full speed):**
☐ **YES** ☐ **NO**

**Assessment:**

**Cleared for Return to Sport?** ☐ **YES** ☐ **NO**

University Hospitals Rehabilitation Services

University Hospitals Orthopedics

# Achilles Repair Guidelines

**Authors:** Chris LeVan, PT, Daniel Chelette, PT, Lauren Bertagnolli, PT

**Reviewed January 2025:** Mario Gastaldo, PT, Diana Gabriel, PT

**Physician Review January 2025:** Shana Miskovsky, MD

**References:**

1. Akizuki KH, Gartman EJ, Nisonson B, Ben-Avi S, McHugh MP. The relative stress on the Achilles tendon during ambulation in an ankle immobiliser: implications for rehabilitation after Achilles tendon repair. Br J Sports Med. 2001;35(5):329-334.

2. Aufwerber S, Heijne A, Edman G, Silbernagel KG, Ackermann PW. Does Early Functional Mobilization Affect Long-Term Outcomes After an Achilles Tendon Rupture? A Randomized Clinical Trial. Orthop J Sports Med. 2020;8(3):2325967120906522.

3. Baxter JR, Corrigan P, Hullfish TJ, O'Rourke P, Silbernagel KG. Exercise Progression to Incrementally Load the Achilles Tendon. Med Sci Sports Exerc. 2021;53(1):124-130.

4. Briggs-Price S, Mangwani J, Houchen-Wolloff L, et al. Incidence, demographics, characteristics and management of acute Achilles tendon rupture: An epidemiological study. PLoS One. 2024;19(6):e0304197.

5. Brumann M, Baumbach SF, Mutschler W, Polzer H. Accelerated rehabilitation following Achilles tendon repair after acute rupture - Development of an evidence-based treatment protocol. Injury. 2014 Nov;45(11):1782-90.

6. Centner C, Jerger S, Lauber B, et al. Similar patterns of tendon regional hypertrophy after low-load blood flow restriction and high-load resistance training. Scand J Med Sci Sports. 2023;33(6):848-856.

7. Centner C, Lauber B, Seynnes OR, et al. Low-load blood flow restriction training induces similar morphological and mechanical Achilles tendon adaptations compared with high-load resistance training. J Appl Physiol (1985). 2019;127(6):1660-1667.

8. Demangeot Y, Whiteley R, Gremeaux V, Degache F. The load borne by the Achilles tendon during exercise: A systematic review of normative values. Scand J Med Sci Sports. 2023;33(2):110-126.

9. Deng S, Sun Z, Zhang C, Chen G, Li J. Surgical Treatment Versus Conservative Management for Acute Achilles Tendon Rupture: A Systematic Review and Meta-Analysis of Randomized Controlled Trials. J Foot Ankle Surg. 2017;56(6):1236-1243.

10. Eliasson P, Agergaard AS, Couppé C, et al. The Ruptured Achilles Tendon Elongates for 6 Months After Surgical Repair Regardless of Early or Late Weightbearing in Combination With Ankle Mobilization: A Randomized Clinical Trial. Am J Sports Med. 2018;46(10):2492-2502.

11. Hansen OB, Papson A, Eble SK, Drakos MC. Effect of Blood Flow Restriction Therapy Following Achilles Rupture and Repair: A Randomized Controlled Trial. Foot Ankle Orthop. 2022 Jan 20;7(1)

University Hospitals
Rehabilitation Services

University Hospitals
Orthopedics

# Achilles Repair Guidelines

**References:**


12. Hoeffner R, Agergaard AS, Svensson RB, et al. Tendon Elongation and Function After Delayed or Standard Loading of Surgically Repaired Achilles Tendon Ruptures: A Randomized Controlled Trial. Am J Sports Med. 2024;52(4):1022-1031

13. Hong JY, Kang C, Kim TG, et al. Risk Factors for Contralateral Tendon Rupture in Patients With Acute Achilles Tendon Rupture. J Foot Ankle Surg. 2023;62(5):779-784.

14. Hsu AR, Jones CP, Cohen BE, Davis WH, Ellington JK, Anderson RB. Clinical Outcomes and Complications of Percutaneous Achilles Repair System Versus Open Technique for Acute Achilles Tendon Ruptures. Foot Ankle Int. 2015;36(11):1279-12

15. Kangas J, Pajala A, Ohtonen P, Leppilahti J. Achilles tendon elongation after rupture repair: a randomized comparison of 2 postoperative regimens. Am J Sports Med. 2007;35(1):59-64.

16. Komi PV, Fukashiro S, Järvinen M. Biomechanical loading of Achilles tendon during normal locomotion. Clin Sports Med. 1992;11(3):521-531.

17. Lantto I, Heikkinen J, Flinkkila T, et al. A Prospective Randomized Trial Comparing Surgical and Nonsurgical Treatments of Acute Achilles Tendon Ruptures. Am J Sports Med. 2016;44(9):2406-2414.

18. LaPrade CM, Chona DV, Cinque ME, et al. Return-to-play and performance after operative treatment of Achilles tendon rupture in elite male athletes: a scoping review. Br J Sports Med. 2022;56(9):515-520.

19. Lee M, Lancaster M, Tulloch L, et al. Normative isometric plantarflexion strength values for professional level, male rugby union athletes. Phys Ther Sport. 2023;61:114-121.

20. Nagelli CV, Hooke A, Quirk N, et al. Mechanical and strain behaviour of human Achilles tendon during in vitro testing to failure. Eur Cell Mater. 2022;43:153-161.

21. Okoroha KR, Ussef N, Jildeh TR, et al. Comparison of Tendon Lengthening With Traditional Versus Accelerated Rehabilitation After Achilles Tendon Repair: A Prospective Randomized Controlled Trial. Am J Sports Med. 2020;48(7):1720-1726.

22. Olsson N, Karlsson J, Eriksson BI, Brorsson A, Lundberg M, Silbernagel KG. Ability to perform a single heel-rise is significantly related to patient-reported outcome after Achilles tendon rupture. Scand J Med Sci Sports. 2014;24(1):152-158.

23. Olsson N, Petzold M, Brorsson A, Karlsson J, Eriksson BI, Silbernagel KG. Predictors of Clinical Outcome After Acute Achilles Tendon Ruptures. Am J Sports Med. 2014;42(6):1448-1455.

24. Pneumaticos SG, McGarvey WC, Mody DR, Trevino SG. The effects of early mobilization in the healing of achilles tendon repair. Foot Ankle Int. 2000;21(7):551-557.

# Achilles Repair Guidelines

**References:**

25. Rendek Z, Bon Beckman L, Schepull T, et al. Early Tensile Loading in Nonsurgically Treated Achilles Tendon Ruptures Leads to a Larger Tendon Callus and a Lower Elastic Modulus: A Randomized Controlled Trial. Am J Sports Med. 2022;50(12):3286-3298.

26. Tarantino D, Palermi S, Sirico F, Corrado B. Achilles Tendon Rupture: Mechanisms of Injury, Principles of Rehabilitation and Return to Play. J Funct Morphol Kinesiol. 2020 Dec 17;5(4):95

27. Valkering KP, Aufwerber S, Ranuccio F, Lunini E, Edman G, Ackermann PW. Functional weight-bearing mobilization after Achilles tendon rupture enhances early healing response: a single-blinded randomized controlled trial. Knee Surg Sports Traumatol Arthrosc. 2017;25(6):1807-1816.

28. Xu XY, Gao S, Lv Y, et al. Duration of immobilisation after Achilles tendon rupture repair by open surgery: a retrospective cohort study. J Orthop Surg Res. 2021;16(1):196.

29. Zellers JA, Baxter JR, Grävare Silbernagel K. Functional Ankle Range of Motion but Not Peak Achilles Tendon Force Diminished With Heel-Rise and Jumping Tasks After Achilles Tendon Repair. Am J Sports Med. 2021;49(9):2439-2446.

30. Zellers JA, Carmont MR, Silbernagel KG. Achilles Tendon Resting Angle Relates to Tendon Length and Function. Foot Ankle Int. 2018;39(3):343-348.

31. Zellers JA, Cortes DH, Silbernagel KG. FROM ACUTE ACHILLES TENDON RUPTURE TO RETURN TO PLAY - A CASE REPORT EVALUATING RECOVERY OF TENDON STRUCTURE, MECHANICAL PROPERTIES, CLINICAL AND FUNCTIONAL OUTCOMES. Int J Sports Phys Ther. 2016;11(7):1150-1159.

32. Zellers JA, Pohlig RT, Cortes DH, Grävare Silbernagel K. Achilles tendon cross-sectional area at 12 weeks post-rupture relates to 1-year heel-rise height. Knee Surg Sports Traumatol Arthrosc. 2020;28(1):245-252.
